# Targeting estrogen/estrogen receptor alpha enhances Bacillus Calmette-Guérin efficacy in bladder cancer

**DOI:** 10.18632/oncotarget.8756

**Published:** 2016-04-15

**Authors:** Zhiqun Shang, Yanjun Li, Iawen Hsu, Minghao Zhang, Jing Tian, Simeng Wen, Ruifa Han, Edward M. Messing, Chawnshang Chang, Yuanjie Niu, Shuyuan Yeh

**Affiliations:** ^1^ Chawnshang Chang Sex Hormone Research Center, Tianjin Institute of Urology, The 2nd Hospital of Tianjin Medical University, Tianjin, China; ^2^ George Whipple Lab for Cancer Research, Departments of Urology, Pathology and the Cancer Center, University of Rochester, Rochester, New York, United States of America

**Keywords:** Bacillus Calmette-Guérin, bladder cancer, estrogen, estrogen receptor alpha

## Abstract

Recent studies showed the potential linkage of estrogen/estrogen receptor signaling with bladder tumorigenesis, yet detailed mechanisms remain elusive. Here we found a new potential therapy with the combination of Bacillus Calmette Guerin (BCG) and the anti-estrogen ICI 182,780 led to better suppression of bladder cancer (BCa) than BCG alone. Mechanism dissection found ICI 182,780 could promote BCG attachment/internalization to the BCa cells through increased integrin-α5β1 expression and IL-6 release, which may enhance BCG-induced suppression of BCa cell growth *via* recruiting more monocytes/macrophages to BCa cells and increased TNF-α release. Consistently, *in vivo* studies found ICI 182,780 could potentiate the anti-BCa effects of BCG in the carcinogen-induced mouse BCa models. Together, these *in vitro* and *in vivo* results suggest that combining BCG with anti-estrogen may become a new therapeutic approach with better efficacy to suppress BCa progression and recurrence.

## INTRODUCTION

Bladder cancer (BCa) is a common malignancy, which resulted in an estimated 74,690 new cases and 15,580 deaths in 2014 in the United States [1]. At initial diagnosis, over 70% of patients have non-muscle-invasive bladder cancers (NMIBC), many of which are treated with transurethral resection of tumor with subsequent intravesical Bacillus Calmette-Guérin (BCG) therapy for elimination of residual tumor cells and prevention of recurrence [2]. However, recurrence rates for NMIBC range from 50% to 70%, and approximately 10-15% of tumours progress to muscle invasive bladder cancer (MIBC) over a 5-year period [2–4]. To date, BCG therapy is the most common intravesical therapy for NMIBC. BCG is a live-attenuated strain of *Mycobacterium bovis* developed in 1921 as a vaccine for tuberculosis. However, it was later found BCG treatment could be beneficial for BCa patients, and the first published report of its use in BCa therapy was in 1976 [5]. Although BCG is the most effective agent currently available for NMIBC, approximately 30% of patients treated with intravesical BCG fail to respond to this agent [6]. Even in the face of an initial response, long-term outcomes suggest a relatively high rate of disease recurrence and/or progression within five years [7, 8]. Although advances have led to improved clinical efficacy and better understanding of the immunologic basis for this therapy since 1976, the mechanism of BCG immunotherapy remains to be further investigated.

Although women have a lower BCa incidence, recent evidence showed that women were more likely to develop to advanced tumor stages with nodal metastasis, and more frequently received chemotherapy compared to the male counterparts [9–12]. Those findings were in line with previous studies, which have shown worse outcomes in women patients compared to men with NMIBC [3] or MIBC [13–15]. Recent studies have found estrogen receptor (ER) expression in BCa tissues and cell lines [16–18].

There are two major types of ERs, ER-alpha (ERα) and ER-beta (ERβ), mediating estrogen effects in various tissues [19–22], and could play important roles in BCa progression [23–26]. Although ERα was reported to have a protective role in BCa initiation and growth [24, 27], however, it remains unclear whether ERα expression and activity could influence BCG therapy response. A previous report showed that estrogen could down-regulate NF-κB mediated IL-6 expression in human BCa lines [28], and IL-6 is one of the cytokines elicited in response to BCG and could up-regulate the cellular expression of integrin-α5β1, the receptor complex on which BCG adherence depends [29–30]. It is suggested that alteration of the autocrine IL-6 response to BCG via pharmacological manipulation of the estrogen milieu may have a therapeutic value for urothelial carcinoma, however, the underlying mechanisms remain unclear. Also, the questions of whether estrogen/ER signals can affect BCG efficacy via (i) influencing the BCG attachment and internalization and (ii) altering the infiltration and the secretion profile of immune cells remain uninvestigated areas. Here, we report how anti-estrogens function via multiple mechanisms to enhance the effect of BCG against urothelial carcinoma.

## RESULTS

### Anti-estrogen potentiates BCG attachment/internalization to the BCa cells

**Figure 1:**
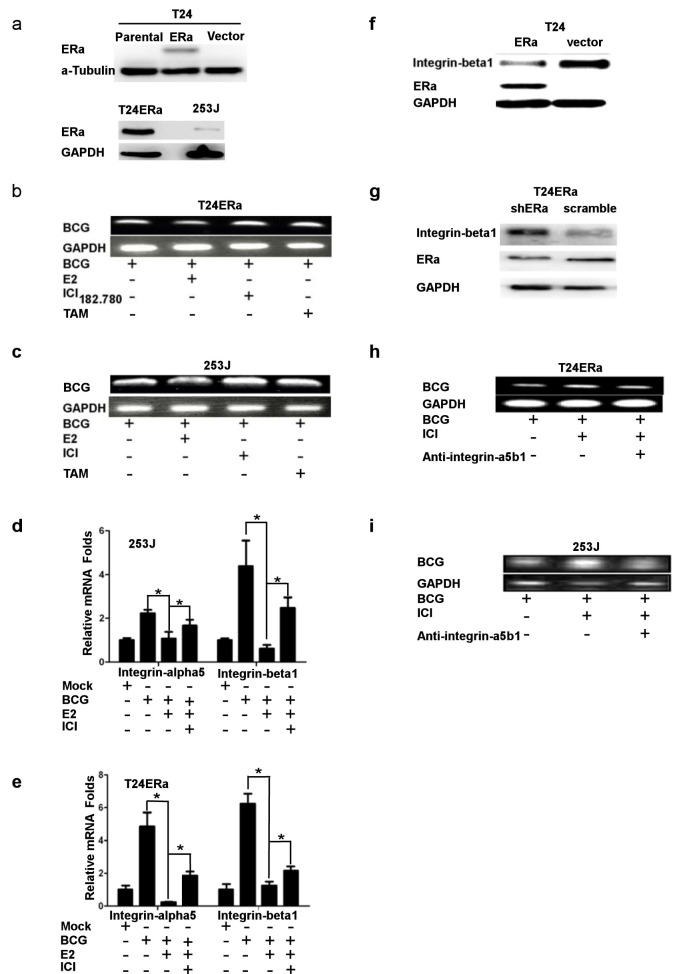
ICI182,780 (ICI) promotes BCG attachment and internalization through regulating integrin-α5β1 pathway in BCa cells **a.** ERα expression in T24ERα and 253J cells. **b.** and **c.** ICI 182,780 increases BCG attachment and internalization. We seeded 4×10^5^T24ERα (b) and 253J (c) cells into the plate. Cells were treated with 1 μM ICI and/or 1 nM E2 for 12 h, then incubated with BCG (2×10^7^CFU) for 2 h. BCG was washed away by 1xPBS for 3 times, and genomic DNA was extracted to perform PCR using the designed primers to detect BCG. **d.** and **e.** The T24ERα (d) and 253J (e) cells were treated under the same experimental conditions as in Figure 1A, RNA was extracted, and mRNA levels of integrin-α5 and β1 were determined using Q-PCR. **f.** and **g.** Western blot was used to detect integrin-beta1 expression. **h.** and **i.** Blocking integrin-α5β1 reduces BCG attachment and internalization. We seeded 4×10^5^ T24ERα (h) and 253J (i) cells into the plate. Cells were treated with mock or 1 μM ICI for 12 h, then incubated with BCG (2×10^7^CFU) and integrin-α5β1 neutralizing antibody for 2 hr. After incubation, unbound BCG was washed out with 1xPBS for 3 times and genomic DNA was collected to perform PCR by using BCG primers. *indicates *p*<0.05.

Early reports suggested that BCG was able to function via attachment/internalization in BCa cells to alter the immune responses and consequently exert its immunotherapeutic effects [31–33]. We therefore decided to use two ERα positive BCa urothelial cell lines (Figure 1a) to investigate the potential effect of anti-estrogen ICI 182,780 on the BCG immunotherapeutic effect *via* influencing BCG attachment/internalization to BCa cells. We first applied PCR to detect BCG internalization in BCa urothelial cells, and found addition of either 1 μM ICI 182,780 or tamoxifen (TAM) significantly increased the BCG internalization (Figure 1b and 1c).

As early reports suggested that BCG could bind to fibronectin and the fibronectin/integrin-α5β1 complex functioned as a bridge complex to promote BCG attachment/internalization to BCa cells, we then examined the E2/ER effects on integrin-α5β1, and its consequence on BCG attachment/internalization to BCa urothelial cells. The results from Figure 1d and 1e clearly demonstrated E2 treatment significantly decreased the integrin-α5β1 mRNA expression in these two BCa cells induced by BCG. Interestingly, ERα can directly down-regulate integrin-α5β1 expression in the two BCa urothelial cells (Figure 1f and 1g). We then added integrin-α5β1 antibody to see if neutralization of integrin-α5β1 could interrupt the ICI 182,780 enhanced BCG attachment/internalization to BCa cells, and results showed the neutralization of integrin-α5β1 could reduce the ability of the ICI 182,780 enhanced BCG attachment/internalization to BCa cells (Figure 1h and 1i). Together, results from Figure 1 demonstrated that anti-estrogen could enhance BCG attachment/internalization to BCa urothelial cells *via* induction of integrin-α5β1 expression.

### Estrogen reduces IL-6 expression that led to less monocytes/macrophages migration toward the BCa cells

**Figure 2:**
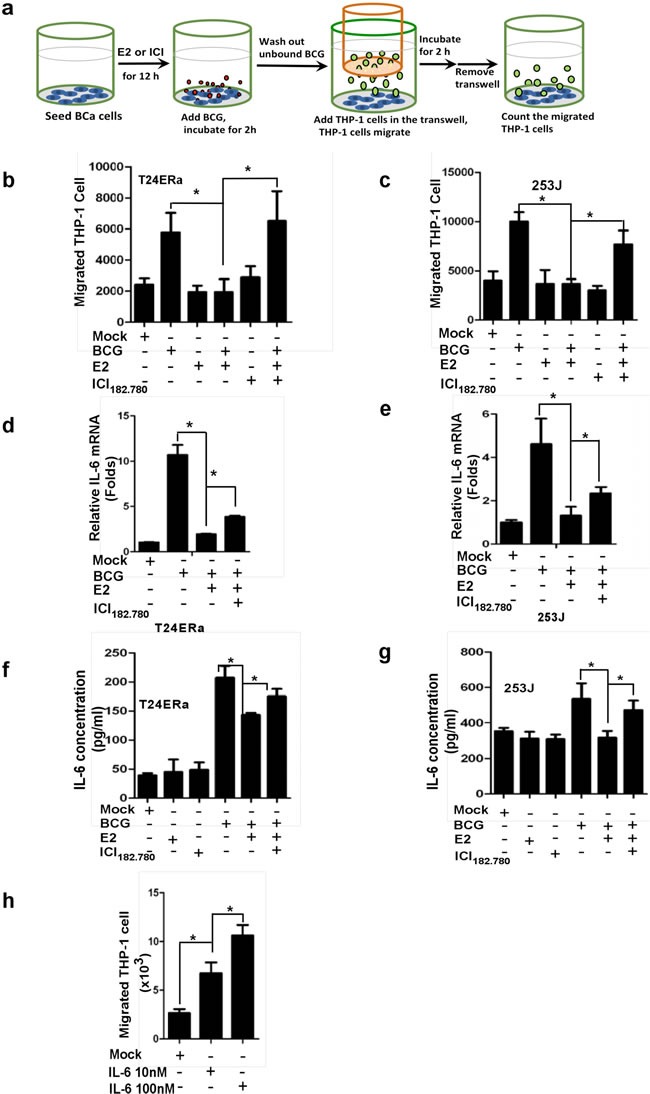
ICI 182,780 (ICI) promotes monocyte/macrophage migration toward BCG-treated BCa cells **a.** 5×10^4^BCa cells were seeded into the bottom chambers of transwells. cells were treated with 1 μM ICI and/or 10nM E2 for 12h and BCG was added and incubated for an additional 2 h. After washing out unattached BCG, inserts were added and 4×10^5^ THP-1 cells were added into the upper well and then co-incubated with BCa cells for 2 h. The media from the bottom well were collected to count the migrated THP1 cells. **b.** and **c.** THP-1 cell migration toward T24 ERα or 253J cells. Each experiment was performed in triplicate. **d.**-**g.** E2 decreased BCG binding and internalization to BCa, led to reduce IL-6 production, and ICI can reverse E2 mediated inhibition. We seeded 4×10^5^ T24 ERα or 253J cells into the plate. Cells were treated with 1 μM ICI in the presence of 1 nM E2 for 12 h, and then incubated with BCG (2×10^7^CFU) for 2 h. After washing away unbound BCG, mRNA was extracted from BCa cells to determine IL-6 expression using Q-PCR. **h.** ICI promotes THP-1 migration through regulating IL-6 level in BCa cells. CMs were collected from BCa cells treated with ICI or E2 and BCG as indicated in the figure design. The 10 nM or 100 nM recombinant human IL6 (rhIL-6) was added into the bottom wells, 4×10^5^THP-1 cells were added into the upper wells, and co-cultured for 2 h. The media from the bottom wells was collected to count the migrated THP1 cells. Each experiment was performed in triplicate. *indicates *p*<0.05.

To further study how ICI 182,780 could enhance the BCG therapy efficacy to suppress BCa, we then applied the co-culture system to examine if ICI 182,780 might affect BCG induced immune responses in BCa cells as early reports suggested that BCG-induced anti-BCa was linked to the alteration of local activity of immuno-competent cells [34]. We seeded BCa T24ERα cells or 253J cells in the bottom wells and monocytes/macrophage THP-1 cells on the top transwells (Figure 2a), and co-cultured cells were treated with or without BCG and/or anti-estrogens. As shown in Figure 2b and 2c, addition of BCG increased THP-1 cells migration to BCa cells, yet E2 treatment significantly reduced THP-1 cells migration to BCa cells. ICI182,780 treatment could reverse the E2-inhibited THP-1 cells migration.

To further dissect the mechanism at the molecular level why estrogen could reduce BCG efficacy on monocytes/macrophages migration toward BCa cells, we then examined the altered immune responses *via* assay of IL6 expression in T24ERα (Figure 2d & 2f) and 253J (Figure 2e & 2g) cells, as early reports suggested that BCG may enhance IL6 expression to elicit its therapeutic effects on BCa [35]. As expected, adding BCG led to the increased IL6 expression in both mRNA and protein level, and addition of E2 could decrease IL-6 expression induced by BCG treatment. Addition of ICI 182,780 reversed the E2 inhibited effect on IL6 expression in both T24ERα and 253J cells (Figure 2d-2g). Importantly, adding neutralizing anti-IL6 antibody resulted in reduction of monocytes/macrophages migration to BCa cells (Figure 2h), showing IL-6 is a key factor for BCa to attract immune cell infiltration toward BCa. Together, results from Figure 2 proved that E2 could decrease IL6 expression in the BCa cells that led to less monocytes/macrophages migration towards BCa cells.

### Increased monocytes/macrophages migration to BCa cells led to more TNF-α secretion to kill more BCa cells

**Figure 3:**
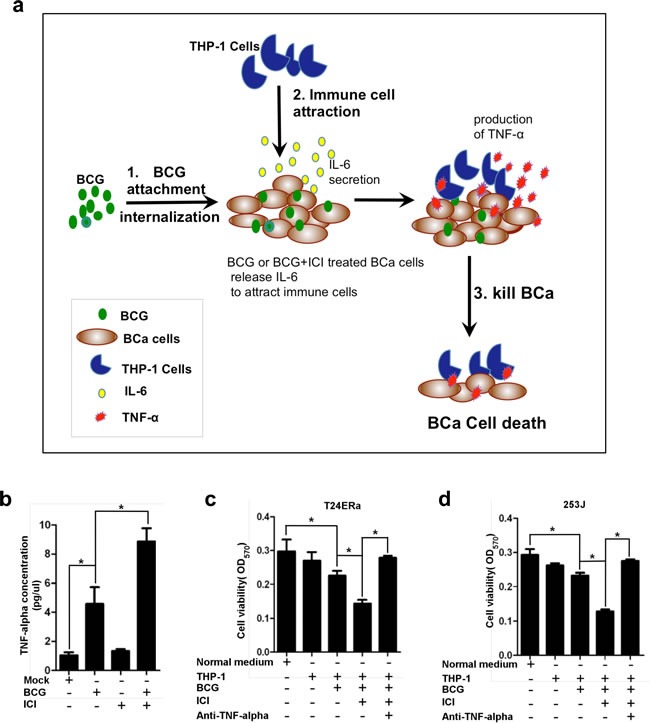
Monocytes/macrophages recruitment to BCa under the BCG plus ICI treatment can more effectively inhibit the BCa cell growth **a.** The schematic mechanism presentation of monocytes/macrophage recruitment induced by BCG or by BCG plus ICI. The increased immune cell infiltration resulted in a higher efficacy to suppress BCa cells. **b.** We co-cultured BCa cells with TPH-1 cells, then treated them with BCG, or BCG plus ICI in normal media for 48 hr. We then collected the conditioned media for detecting TNF-α protein level by ELISA assay. **c.** and **d.** 1x 10^6^ THP-1 cells were cultured in 6 well plates, treated with BCG only, BCG+ICI, PBS only, or ICI only. CMs from these 4 different treatment groups were collected to treat BCa cells for 72 h. The T24ERα (c) and 253J (d) cell viability were detected by MTT assay. * indicates *p*<0.05.

Next, we asked whether recruiting monocytes/macrophages to BCa after combined treatment of BCG and ICI 182,780 could lead to higher efficacy in killing BCa cells. A recent study suggested that BCG may function through recruitment of macrophages to suppress BCa that involved in higher amount of macrophage released soluble cytotoxic factors, including TNF-α, IFN-γ and NO [36]. We co-cultured BCa cells with TPH-1 cells, then treated them with BCG, or BCG plus ICI 182,780 in normal media for 48 hr. We then collected the media for detecting TNF-α protein level by ELISA assay. The results showed that TNF-α protein level in ICI 182,780 plus BCG treated group was higher than other groups (Figure 3b). Subsequently, we collected different group's conditioned medium (CM) to treat BCa cells. We found that CM collected from cells under the BCG plus ICI 182,780 treatment has a higher level of TNF-α (Figure 3b, lane 4), and could more effectively inhibit T24ERα and 253J cell viability (Figure 3c and 3d). As expected, addition of anti-TNF-α antibody then interrupted monocytes/macrophages/BCG/ICI inhibited BCa cell viability (Figure 3c and 3d).

Together, results from Figure 3 suggest that ICI 182,780 enhanced BCG efficacy to kill more BCa cells, which might function through attracting more monocytes/macrophages that result in the increase of TNF-α levels.

### ICI 182,780 potentiates the anti-BCa effects of BCG in BBN-induced mouse BCa model

We applied the mouse BCa model to prove the above *in vitro* findings. 12-weeks-old FVB female mice were divided into 4 group (10 mice per group), fed with water containing 0.05% BBN for 12 weeks. Each group of mice were then injected with 1) vehicle control, 2) BCG alone (2×10^6^cfu/mouse, intravesical injection weekly for 4 weeks), 3) ICI 182,780 alone (0.1 mg/kg body weight, i.p. injected every other day for 4 weeks) and 4) BCG (2×10^6^ CFU/mouse, intravesical injection weekly for 4 weeks)+ ICI 182,780 (0.1 mg/kg body weight, i.p. injected every other day for 4 weeks). Mice were then sacrificed 48 hr after the last injection and bladders were collected for collected for further examination.

**Figure 4:**
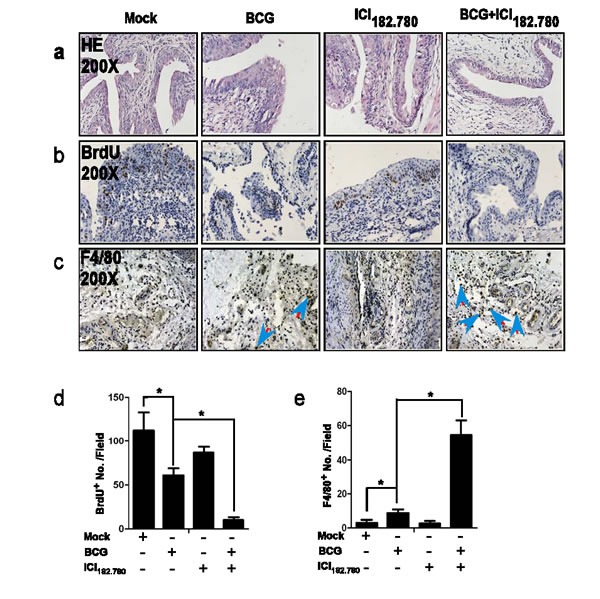
ICI 182,780 (ICI) potentiates the anti-tumor effects of BCG in the BBN-induced mouse BCa model Twelve weeks old FVB female mice were divided into 4 groups (10 mice per group) and treated with 0.05% BBN in drinking water for 12 weeks. Groups (1-4) mice were then injected with 1) mock control, 2) BCG alone (2×10^6^CFU/mouse, intravesical injection weekly), 3) ICI alone (0.1 mg/mouse, i.p. injected every other day,) and 4) BCG (2×10^6^CFU/mouse, intravesical injection weekly) plus ICI (0.1 mg/mouse, i.p. injected every other day). day). At 24 hr after the last treatment, mice were injected with BrdU. At 24 hr after the BrdU injection, mice were then sacrificed and bladders were collected for further examination. **a.** H&E staining. **b.** BrdU Immunohistostaining for BCa cells proliferation. **c.** We used monocyte/macrophage marker (F4/80) antibody to assay monocyte/macrophage cell infiltration. **d.** and **e.** Quantitation shows the positively stained cell numbers per field of BrdU and F4/80, respectively. Significance was defined as *p < 0.05 by student *t* test.

Using HE staining (Figure 4a), we found mice that received vehicle injection or ICI 182,780 injection developed bladder papilloma and carcinoma in situ, and BCG treated mice developed papillonodular hyperplasia. Importantly, mice injected with both BCG plus ICI 182,780 just only developed simple hyperplasia, suggesting ICI 182,780 indeed enhanced BCG efficacy to prevent BCa development in the BBN-induced mouse BCa model.

In order to apply the BrdU staining to assay the proliferation in this BBN-induced BCa mouse model, we injected all mice with BrdU 24 hr prior to euthanization. We found mice treated with BCG alone or ICI 182,780 alone had a reduced BrdU staining compared to mice that received vehicle control, and mice treated with both BCG and ICI 182,780 had the least BrdU staining (Figure 4b and 4d). Together, the above findings confirmed that ICI 182,780 could enhance BCG efficacy to suppress BCa cell proliferation in the BBN-induced BCa mouse model.

Furthermore, we examined the recruitment of macrophages in these BBN-induced mouse BCa model with anti-macrophage F4/80 antibody. Data showed BCG alone helped to recruit more macrophages to BCa, and importantly, BCG plus ICI 182,780 recruited many more macrophages to BCa (Figure 4c and 4e), which is in agreement with above *in vitro* co-culture system (Figure 2) showing ICI 182,780 could enhance BCG to recruit more macrophages to BCa.

Together, results from BCa mouse model clearly demonstrated that ICI 182,780 could enhance BCG efficacy to suppress BCa development and growth.

## DISCUSSION

Intravesical BCG is an effective immunotherapy that, together with endoscopic resection, represents the current standard treatment for patients with high risk NMIBC. Nevertheless, up to 30% of patients fail to respond to an induction course of BCG, while reporting long-term progression rates after treatment have approached 30-50% [29]. Therefore, it is important to identify new strategies to improve the BCG efficacy for those patients at the highest risk of recurrence after the traditional BCG therapy, or for NMIBC patients who are not likely to benefit from a conservative treatment approach. Although women with Ta stage tumors are more likely to receive BCG therapy than men, the importance of hormone status on the outcomes of patients with NMIBC has been less well defined. The immune effect of BCG depends on the BCG binding to the bladder cells. In particular, it has been shown that BCG adherence depends upon the integrin-α5β1 receptor complex and that this complex is up-regulated by the cytokine IL-6 [29]. Interestingly, IL-6 expression is inhibited by estrogen, which has been shown to reverse this effect [28]. Thus, as blocking the attachment of BCG has previously been shown to inhibit the effectiveness of BCG in preventing intravesical tumor growth [37], there is the potential that the hormonal milieu might affect the efficacy of BCG antitumor treatment.

In this study, we identified that estrogen could reduce BCG-induced immunotherapeutic function against urothelial carcinoma by down-regulating the expression of IL-6, integrin-α5β1 and TNF-α, which are the key molecules to mediate BCG-induced monocyte/macrophage recruitment and kill BCa cells, and importantly, ICI 182,780 could potentiate the function of BCG *via* increasing these key molecules’ expressions *in vitro* and *in vivo*. Our current findings showing the combinational therapy of BCG with anti-estrogen drugs, such as ICI 182,780 leads to better efficacy to suppress BCa progression may allow the development of a potential new strategy to battle BCa in those patients with higher recurrence rate.

In summary, estrogen/ER signaling may contribute to refractoriness or the reduced response to BCG. Our findings show the combination of BCG with ICI 182,780 could lead to better suppression of BCa recurrence and progression, and may provide BCa patients a new and better therapy in the near future.

## MATERIALS AND METHODS

### Reagents

ICI 182,780 and E2 were obtained from Sigma (Madison, WI). Polyclonal antibodies against F4/80 and integrin-α5β1 were obtained from Abcam. Anti-BrdU monoclonol antibody came from BD Biosciences (San Jose, CA). The liquid DAB+ substrate chromogen system-horseradish peroxidase used for immunocytochemistry was obtained from Dako Cytomation (Carpinteria, CA). Penicillin, streptomycin and fetal bovine serum (FBS) were obtained from Invitrogen (Carlsbad, CA). Tris, glycine, NaCl, SDS, bovine serum albumin, and monoclonal antibody against β-actin were obtained from Sigma (Madison, WI).

### Cell lines and culture conditions

The T24ERα cell line was cultured in Hyclone McCoy's 5A medium supplemented with 10% FBS. Human BCa cell line 253J was generously provided by Dr. Colin Dinney (Department of Urology, The University of Texas M.D. Anderson Cancer Center), cultured in T medium supplemented with 10% FBS. The human THP-1 cell line was obtained from the American Type Culture Collection, cultured in RPMI 1640 supplemented with 10% heat inactivated FBS, vitamins, sodium pyruvate, L-glutamine, nonessential amino acids, and penicillin-streptomycin.

### Animals

FVB female mice were obtained from the Jackson Lab (Bar Harbor, Maine). The animals were housed four per cage in a specific pathogen-free animal facility and fed with regular chow diet with water ad libitum. Animal protocols and usage were approved by the University of Rochester Medical Center. Committee on Animal Resources, and the mice were housed in the Vivarium of the University of Rochester Medical Center.

### RNA extraction and qPCR analysis

Total RNAs were extracted by Trizol reagent (Invitrogen, Carlsbad, CA) according to the manufacturer's instructions. For RT-PCR, 1 μg of total RNA was reverse-transcribed using the iScript synthesis kit (Bio-Rad, Hercules, CA), according to the manufacturer's protocol and our previous publications [38, 39]. The sequence of primers used in the RT-PCR and qPCR was as followed: human β-actin: 5′-ATC TGG CAC CAC ACC TTC TA-3′ (F), and 5′- CGT CAT ACT CCT GCT TGC TG-3′ (R); human GAPDH: 5′-GCT CTC CAG AAC ATC ATC C-3′ (F), and 5′-TGCTTCACCACCTTC TTG-3′ (R); human integrin-α5 5′-CCT GGC TGG CTG GTA TTA GC-3′ (F) and 5′-GTC GGG GGC TTC AAC TTA GAC-3′ (R); human integrin-β1: 5′-TTA TTG GCC TTG GAT TAC TGC T-3′ (F) and 5′-CCA CAG TTG TTA CGG CAC TCT-3′ (R); BCG: 5′-CCT GCG AGG GTA GGC GTC GG-3′ (F), and 5′-CTC GTC CAG CGC CGC TCC GG-3′ (R). Expression levels were normalized to the expression of GAPDH mRNA.

### MTT assay

The anti-proliferative effects of ICI 182,780 on BCG treated BCa cell lines, 253J and T24ERα, were determined by MTT assay. Cells were plated onto 24-well plates. At various time points indicated, MTT solution (Promega, Madison, WI) was added onto cells for 2 hr, then media were removed, DMSO was used to dissolve the MTT salt, and ODs were measured at 570 nm.

### BCG attachment and internalization assay

We used PCR to detect BCG attachment and internalization. After incubating and washing out non-attached BCG, the cell monolayers were washed twice with Hanks BSS (Gibco). Cells were then harvested using Cell Disassociation Solution (Sigma). Genomic DNA was extracted according to manufacturer's instruction of the Dneasy Blood and Tissue kit (QiaGen).

### Migration assay

5×10^4^BCa cells were seeded into the bottem wells (5μm pore size transwell, Corning Incorporated), pretreated with ICI 182,780 for 12 hr, BCG treated for 2 hr, then BCG was washed out, and 4×10^5^THP-1 cells were added into the upper transwell, and incubated for 2 hr. THP-1 cells that moved to the bottom wells were collected and counted under microscope.

### Western blot analysis

Harvested cells were washed with PBS and lysed in RIPA buffer (50 mM Tris-HCl/pH 7.4; 1% NP-40; 150 mM NaCl; 1 mM EDTA; 1 mM PMSF; 1 mM Na_3_VO_4_; 1 mM NaF; 1 mM okadaic acid; and 1 mg/ml aprotinin, leupeptin, and pepstatin). Individual samples (30 μg protein) were separated on 8-10% SDS-PAGE gel and transferred to PVDF membranes (Millipore, Billerica, MA). Membranes were blocked in a PBST solution with 5% fat-free milk for 1 hr at room temperature, and then the membranes were incubated with appropriate dilutions of specific primary antibodies overnight at 4°C. After washing, the blots were incubated with HRP conjugated anti-rabbit or anti-mouse IgG for 1 hr. The blots were developed in ECL mixture (Vector Lab, Burlingame, CA) and visualized by Imager.

### ELISA assay

We detected IL-6 and TNF-α in the conditioned medium (CM) by enzyme-linked immunosorbent assay (ELISA). BCa cells were treated with different treatments (as indicated in the figure), changed to fresh media and cultured for 24 hr before the media were collected. Human IL-6 and TNF-α ELISA kits (eBioscience) were used to measure IL-6 and TNF-α concentration following the manufacturer's instructions.

### N-butyl-N-(4-hydroxybutyl) nitrosamine (BBN)-Induced Mouse Bladder Cancer Model

12 weeks old FVB female mice (Jackson Lab) were supplied ad libitum with tap water containing 0.05% BBN (TCI America, Portland, OR) in opaque bottles for a total of 12 weeks and thereafter with tap water without BBN. The drinking water was prepared fresh twice a week, and consumption was recorded to estimate BBN intake. Then mice were randomly divided into 4 groups (10 mice per group) as soon as mouse urine tested positive for blood. Four groups of mice were then treated with (i) mock control, (ii) BCG alone (2×10^6^CFU/mouse, intravesical injection weekly), (iii) ICI 182,780 alone (0.1 mg/kg body weight, i.p. injected every other day), and (iv) BCG (2×10^6^ CFU/mouse, intravesical injection weekly)+ ICI 182,780 (0.1 mg/kg body weight, i.p. injected every other day). Mice were then sacrificed 48 hr after the last treatment, and bladder were then collected for further examination.

### Immunohistochemistry

Mice were injected with BrdU reagent 24 hr before sacrificed. Formalin-fixed, paraffin-embedded bladder tissue sections were stained with anti-BrdU (mouse monoclonal clone; BD) and anti-F4/80. Results were expressed as average ± SD of positive cells per ×200 magnification field. A total of six ×200 fields were examined and counted from each group.

### Statistical analysis

Values were expressed as mean ± standard deviation (S.D.). The Student's *t* and ANOVA tests were used to calculate P values. P values were two-sided, and considered statistically significant when <0.05.

## SUPPLEMENTARY MATERIALS FIGURES


