# Supplementary material for: Targeting estrogen/estrogen receptor alpha enhances Bacillus Calmette-Guérin efficacy in bladder cancer
Source: Oncotarget. 2016 Apr 15;7(19):27325–35. doi: 10.18632/oncotarget.8756 (PMC5053653; doi:10.18632/oncotarget.8756)
Supplement: Supplementary file 1 [file oncotarget-07-27325-s001.pdf]

Targeting estrogen/estrogen receptor alpha will enhance Bacillus Calmette-Guerin efficacy in bladder cancer

Supplementary Material

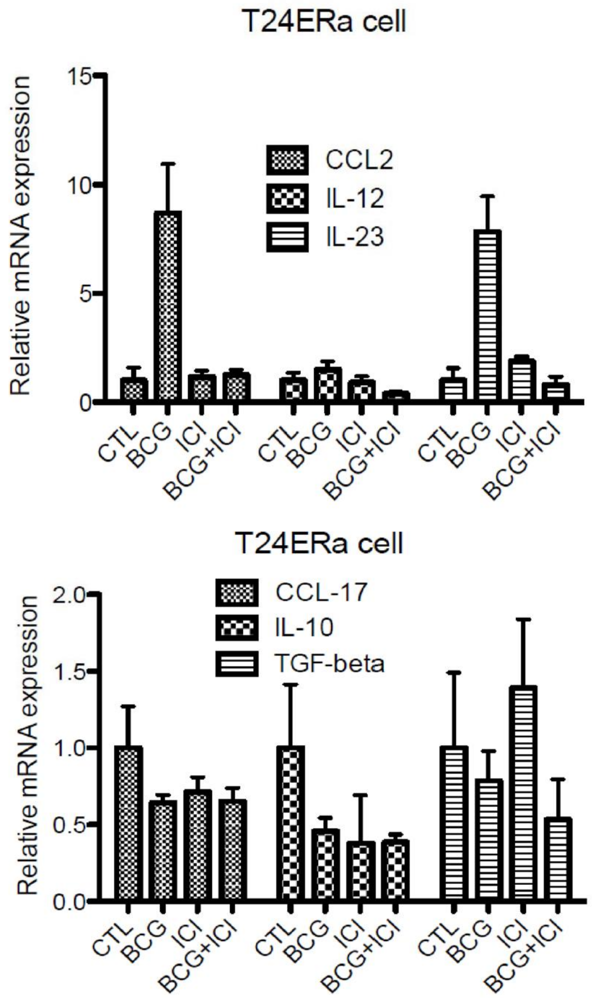

Supplemental figure ICI$_{182.780}$ promotes THP-1 cell differentiating to M1 macrophage induced by BCG

5x10^4 T24ERα cells were seeded into the bottom of transwell and pre-treated with ICI for 12 h, then BCG was added and incubated for additional 2 h. After washing out BCG, RPMI1640 was changed to the well and incubated for 24 hour, then collected condition medium. THP-1 cell was incubated with the above condition medium for 48hr. Then, the total RNA was extracted from THP-1 cell to determine CCL2, IL-10,IL-23,TNF-β,CCL17 and IL-12 expression using qPCR assay. Each experiment was performed in triplicates.
